# Accelerometer-measured physical activity in mid-age Australian adults

**DOI:** 10.1186/s12889-022-14333-z

**Published:** 2022-10-21

**Authors:** Gregore Iven Mielke, Nicola W Burton, Wendy J Brown

**Affiliations:** 1 School of Public Health, The University of Queensland, 4006 Brisbane, QLD Australia; 2 Menzies Health Institute, Griffith University, Gold Coast, Australia; 3 Centre for Mental Health, Griffith University, Brisbane, Australia; 4 School of Human Movement and Nutrition Sciences, The University of Queensland, Brisbane, Australia; 5 Faculty of Health Sciences and Medicine, Bond University, Gold Coast, Australia; 6 School of Applied Psychology, Griffith University, Brisbane, Australia

**Keywords:** Physical activity monitor, Cohorts, Adulthood, Accelerometer, GGIR, Devices

## Abstract

**Background:**

Raw data from accelerometers can provide valuable insights into specific attributes of physical activity, such as time spent in intensity-specific activity. The aim of this study was to describe physical activity assessed with raw data from triaxial wrist-worn accelerometers in mid-age Australian adults.

**Methods:**

Data were from 700 mid-age adults living in Brisbane, Australia (mean age: 60.4; SD:7.1 years). Data from a non-dominant wrist worn triaxial accelerometer (Actigraph wGT3X-BT), expressed as acceleration in gravitational equivalent units (1* mg* = 0.001* g*), were used to estimate time spent in moderate-vigorous intensity physical activity (MVPA; >100 m*g*) using different bout criteria (non-bouted, 1-, 5-, and 10-min bouts), and the proportion of participants who spent an average of at least one minute per day in vigorous physical activity.

**Results:**

Mean acceleration was 23.2 *mg* (SD: 7.5) and did not vary by gender (men: 22.4; women: 23.7; p-value: 0.073) or education (p-value: 0.375). On average, mean acceleration was 10% (2.5 m*g*) lower per decade of age from age 55y. The median durations in non-bouted, 1-min, 5-min and 10-min MVPA bouts were, respectively, 68 (25th -75th : 45–99), 26 (25th -75th : 12–46), 10 (25th -75th : 3–24) and 8 (25th -75th : 0–19) min/day. Around one third of the sample did at least one minute per day in vigorous intensity activities.

**Conclusion:**

This population-based cohort provided a detailed description of physical activity based on raw data from accelerometers in mid-age adults in Australia. Such data can be used to investigate how different patterns and intensities of physical activity vary across the day/week and influence health outcomes.

## Introduction

Because of the importance for public health, global surveillance of physical activity has improved substantially in recent years [1]. Data from the Global Observatory for Physical Activity show that at least 90% of countries worldwide have estimates of self-reported physical activity from at least one survey, and approximately 30% maintain physical activity surveillance at the population level [2].

These self-reported data are important because they have demonstrated associations with numerous health outcomes [3]. The typical questions used to assess physical activity assess structured and purposive behaviour, mostly in relation to active transport and recreational activities [4]. Time spent in physical activities which are more ‘incidental’ (for example short episodes of walking during paid or unpaid work, or taking the stairs) are not be captured in most self-report measures [4]. This is important because these ‘incidental’ activities may also be associated with positive health outcomes and may vary by sociodemographic characteristics [3, 5].

The use of accelerometry in population-based studies has increased in recent years and has created windows of opportunity for researchers to assess the full spectrum of physical activities [5–7]. These studies have confirmed the prolific self-report data which show that time spent in moderate-to-vigorous physical activity is beneficial for health [3, 5, 8]. Recent studies with accelerometry-measured physical activity have also shown that the protective effect for all-cause mortality [8] and specific health outcomes related to physical function [9] may be considerably larger than those observed in studies of self-report measures of physical activity. There is also growing evidence to suggest that light intensity activity, and even small amounts of high-intensity habitual physical activity, may be beneficial for health [10, 11]. Stiles and colleagues have shown that pre-menopausal women who spent just one minute per day doing very high intensity physical activity (equivalent to running), had better bone health than their counterparts who did not [11]. Stamatakis et al., have also suggested that high intensity ‘incidental’ activity (such as running up the stairs) may be beneficial for improving health among adults with low levels of physical activity [12].

Accelerometer data provide numerous metrics for describing physical activity in terms of patterns of movement at different intensities and in different bout durations. Furthermore, open-access codes that can be used to convert raw accelerometry data into estimates of physical activity, in a variety of intensities and durations, has enabled comparability between studies without using brand-specific count cut-points. [13] This may help to improve understanding of sociodemographic and other determinants of physical activity, which will help to identify key intervention points and target groups, as well as associated health outcomes. [14–16]. The overall aim of this study was to describe physical activity assessed with raw data from triaxial wrist-worn accelerometers in mid-age Australian adults. The specific aims were to [1] describe daily acceleration, as an indicator of overall physical activity in mid-age Australian adults; [2] compare time spent in bouts of differing duration of moderate-vigorous physical activity (MVPA); [3] compare differences in time spent in non-bouted and 10-minute bouted MVPA by gender, age, education, income and occupation; and [4] describe time spent in vigorous-intensity physical activity according to gender, age, education, income and occupation.

## Methods

We analysed data from the HABITAT study [17]. This was a population-based cohort study of mid-age adults living in Brisbane, Australia. A multi-stage sampling process was used to select a representative and socioeconomically diverse sample of over 17,000 adults aged 40–65 years. The baseline measures were collected in 2007 via a mail survey, with 11,085 responses (response rate 68.4%). At baseline in 2007, the sample was representative of the Brisbane population, but with slightly more women, tertiary educated and higher income participants. The participants were surveyed by mail again in 2009, 2011 and 2013. For the present study, 767 people who had responded to the four mail surveys were randomly selected and included in a sub study in 2014 to collect objective measures of physical activity and physical functioning. The mail survey and all study protocol received ethical clearance from the Queensland University of Technology Human Research Ethics Committee (Ref. Nos. 3967 H & 1300000161). The sub study received ethical clearance from the University of Queensland Human Research Ethics Committee (Ref. No. 2013000443). All individual participants were consulted, clarified and accepted participation in the study by signing of an Informed Consent Form. Detailed information on the design of the HABITAT study has been published previously [17–19].

All participants were asked to wear a triaxial accelerometer (Actigraph wGT3X-BT) on the non-dominant wrist during waking hours for seven consecutive days.[20] The accelerometer recorded raw acceleration in three axes and provided raw data expressed in gravitational equivalent units (*g*) (1 g = 9.81 m/s^2^). Data were collected at 30 Hz time resolution. Raw data were processed in R using the most up to date GGIR package, a widely used open-source code [14]. This involved a calibration to local gravity [21, 22], adjustment for non-wear time and a filter for abnormally high values. Non-wear time was defined as periods of at least 60 consecutive minutes of low acceleration with little variability [14]. The vector magnitude of the three axes was used to calculate activity-related acceleration using Euclidian Norm minus 1* g* [ENMO=√(x^2^ + y^2^ + z^2^)-1]. For segments with invalid data, the average of similar time-of-day data points from other days of measurement in the same individual were imputed. Data were initially aggregated in 5-second time series. Data were included if wear time was at least 600 min/day on at least four days. These definitions have been widely used in previous studies with accelerometers.[5].

Data were used to quantify overall physical activity expressed as acceleration in milligravity units (m*g*), as well as time spent in activities at different intensities using intensity thresholds (moderate intensity: acceleration 100–400 m*g*; vigorous intensity: acceleration higher than 400 m*g*) similar to proposed by Hildebrand et al. [23]. Durations of moderate-vigorous physical activity (MVPA) were estimated using four different criteria for bout duration (non-bouted, 1-, 5- and 10-minute bouts). Bouts of physical activity were identified as time windows with activities that started with a 5-s epoch value equal to or higher than the intensity threshold (100 m*g* for moderate; 400 m*g* for vigorous) and for which 80% of subsequent 5-s epoch values were equal to or higher than the intensity threshold. This approach has been widely used in previous studies with raw data from accelerometers [5, 9, 14, 24, 25]. We focused mainly on non-bouted MVPA and bouts lasting at least 10 min, as recommended in previous physical activity guidelines [3].

Sociodemographic characteristics were assessed in 2014 (gender and age) and in 2013 (education, income, and current occupation type) using standardised questionnaire items. Data were categorised as follows: gender (men; women); age (45–54; 55–64; ≥ 65 years); education (year 12 or less; certificate or diploma; bachelor degree or higher); annual household income before tax (< $ 41,599; $41,600 - $93,599; $93,600), and current occupation type (managers or professionals; clerical or administrative; community, personal or sales; labourer; retired).

Statistical analyses were conducted using Stata 17.0. Descriptive analyses were used to summarise physical activity variables according to gender, age, education, income, and occupation. Mean and standard deviation were used to describe overall daily acceleration and minutes in non-bouted MVPA. Minutes in bouts of 10 min were described for MVPA using daily median and interquartile range due to the skewed distribution of the variable. We also estimated the proportion of participants who spent an average of at least one minute per day in vigorous physical activity (VPA). Crude and adjusted regression models were used to investigate the associations of acceleration, non-bouted MVPA, 10 min-bouts of MVPA, and the proportion who reported any VPA, with sociodemographic variables. Due to differences in data distribution, linear regression models were used for acceleration and MVPA (non-bouted), quantile regression for MVPA (10-min bout) and Poisson regression with adjustment for robust variance for any VPA. Adjusted models included mutual adjustment for gender, age groups, education, income and occupation.

## Results

Of the 767 people who consented to participate, 715 participants (93%) wore an accelerometer, and 700 had at least four valid days of measurement (600 + minutes of measurement each day); 80% of participants wore the accelerometer for 7 days. Of the 3,926 days of valid measurement, average wear time was 16.0 h/day (SD: 2.1). Wear compliance was similar across sociodemographic groups. Sensitivity analyses that included only participants who wore the accelerometer for 7 days/10 + hours were conducted, and the results were unchanged. The analytical sample included 60% women and 41% had a university degree; one third of participants were retired (Table 1). The average age was 60.3 (SD: 7.0) years.

**Table 1:** Sample description and average acceleration (m*g*) by sociodemographic characteristics. Brisbane 2016 (N = 700)

Variables	Accelerometry data			Average acceleration (m*g*)						
	**N**	**%**	**Mean (SD)**		**p value** ^**a**^		**β** ^**b**^_**Crude**_ **(95%CI)**		**β** ^**b**^_**Adjusted**_ **(95%CI)** ^**c**^	
Gender					0.902					
Men	282	40.3	23.8 (8.2)				Ref		Ref	
Women	418	59.7	23.8 (7.4)				-0.1 (-1.2; 1.1)		1.0 (-0.3; 2.3)	
Age					< 0.001					
45–54	181	25.9	26.7 (8.6)				Ref		Ref	
55–64	292	41.7	23.9 (7.6)				-2.8 (-4.2; -1.4)		-2.8 (-4.2; -1.3)	
65+	227	32.4	21.3 (6.1)				-5.5 (-6.9; -4.0)		-4.5 (-6.4; -2.7)	
Education					0.525					
Year 12 or less	213	30.5	23.6 (7.4)				Ref		Ref	
Certificated/diploma	204	29.2	24.3 (8.4)				0.6 (-1.8; 2.1)		0.1 (-1.4; 1.7)	
Bachelor degree or higher	281	40.3	23.5 (7.5)				-0.1 (-1.5; 1.3)		-0.2 (-1.8; 1.4)	
Income (per year)					0.002					
< $ 41,599	149	23.2	21.6 (6.9)				Ref		Ref	
$41,600 - $93,599	232	36.1	24.1 (7.9)				2.5 (1.0; 4.1)		0.7 (-0.9; 2.4)	
$93,600 +	262	40.7	24.7 (7.8)				3.1 (1.6; 4.7)		1.0 (-0.9; 2.8)	
Occupation ^bd^					< 0.001					
Managers/ professionals	260	38.7	24.5 (7.8)				Ref		Ref	
Clerical/ administrative	71	10.6	23.3 (6.5)				-1.3 (-3.2; 0.7)		-1.5 (-3.7; 0.7)	
Community/ personal/ sales	68	10.1	26.5 (7.0)				1.9 (-0.1; 3.9)		1.8 (-0.5; 4.0)	
Labourer/technician	66	9.8	28.4 (9.1)				3.8 (1.8; 5.8)		5.2 (2.9; 7.5)	
Retired	158	23.5	20.8 (6.3)				-3.7 (-5.2; -2.3)		-1.3 (2.9; 7.5)	
Other/non paid work	49	7.3	22.6 (7.7)				-1.9 (-4.2; 0.4)		-1.9 (-4.5; 0.6)	

^a^ p-values for the comparison of average daily acceleration between groups

^b^ beta values represent the mean difference in average daily acceleration between groups (compared with the reference group)

^c^ mutually adjusted for other variables in the model

^d^ Labourer/technician: Technicians, trade, machinery operators, drivers and labourers

Values of daily mean acceleration by sociodemographic characteristics are presented in Table 1. Daily mean acceleration was 23.2 m*g* (SD: 7.5). Overall, average daily acceleration did not vary by gender or education. Mean acceleration was lowest in participants who were older, had low income and those who were retired. In the adjusted analyses, age, income and occupation were associated with average daily acceleration. Average acceleration was 5.5 m*g* lower in participants aged 65 + years than those aged 45-54y, 3.1 m*g* higher in those in the top than those in bottom income category, and 5.5 m*g* higher among labourers than among managers and professionals.

**Fig. 1:**
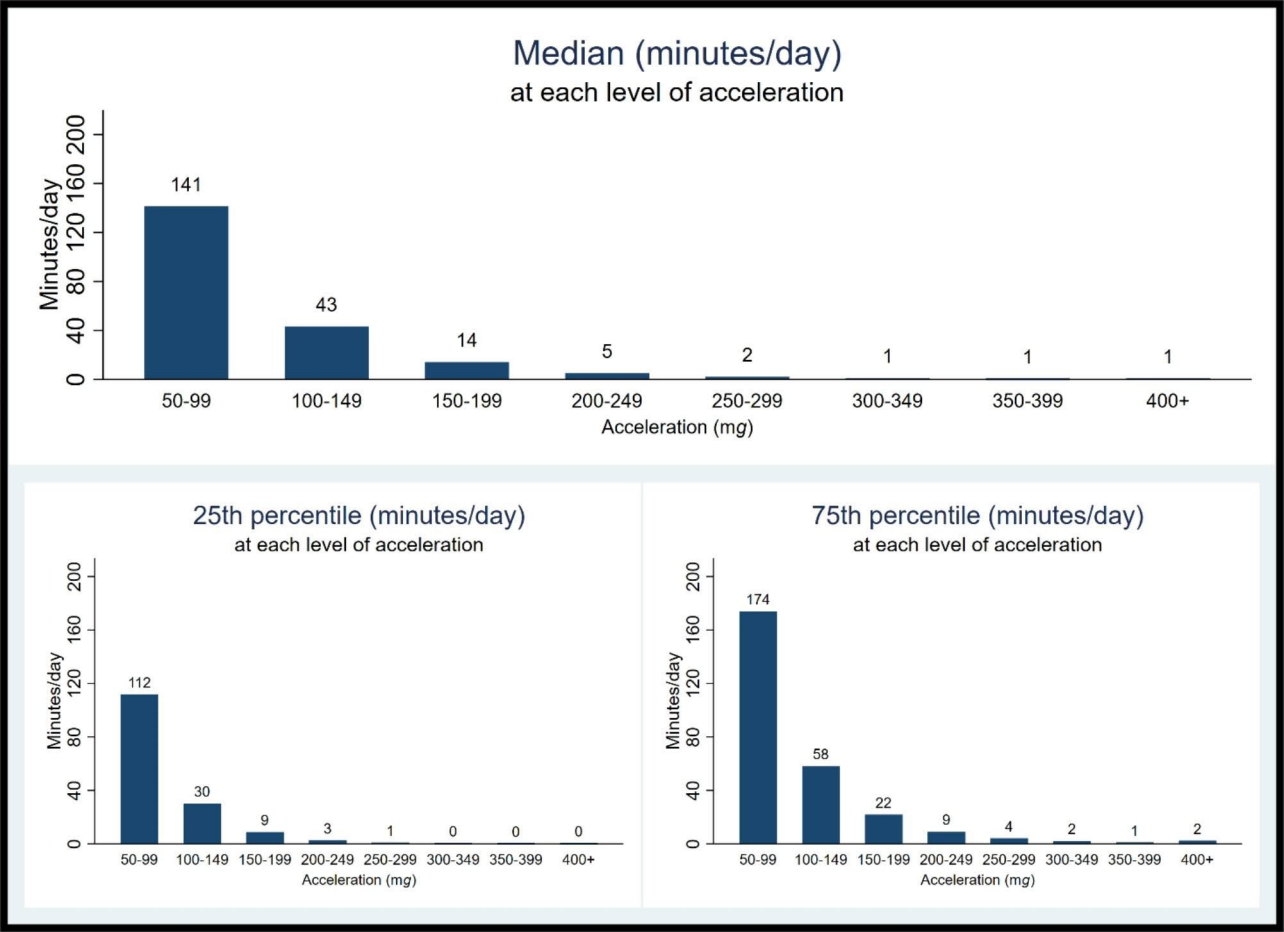
Distribution of daily physical activity (minutes/day) at each level of acceleration. Brisbane 2016 (N = 700). Data should be interpreted as e.g.: sample median of daily minutes spent in activities with acceleration between 50–99 m*g* was 141; sample 25th and 75th percentiles spent in activities with acceleration between 50–99 m*g* were 112 and 174, respectively

**Fig. 2:**
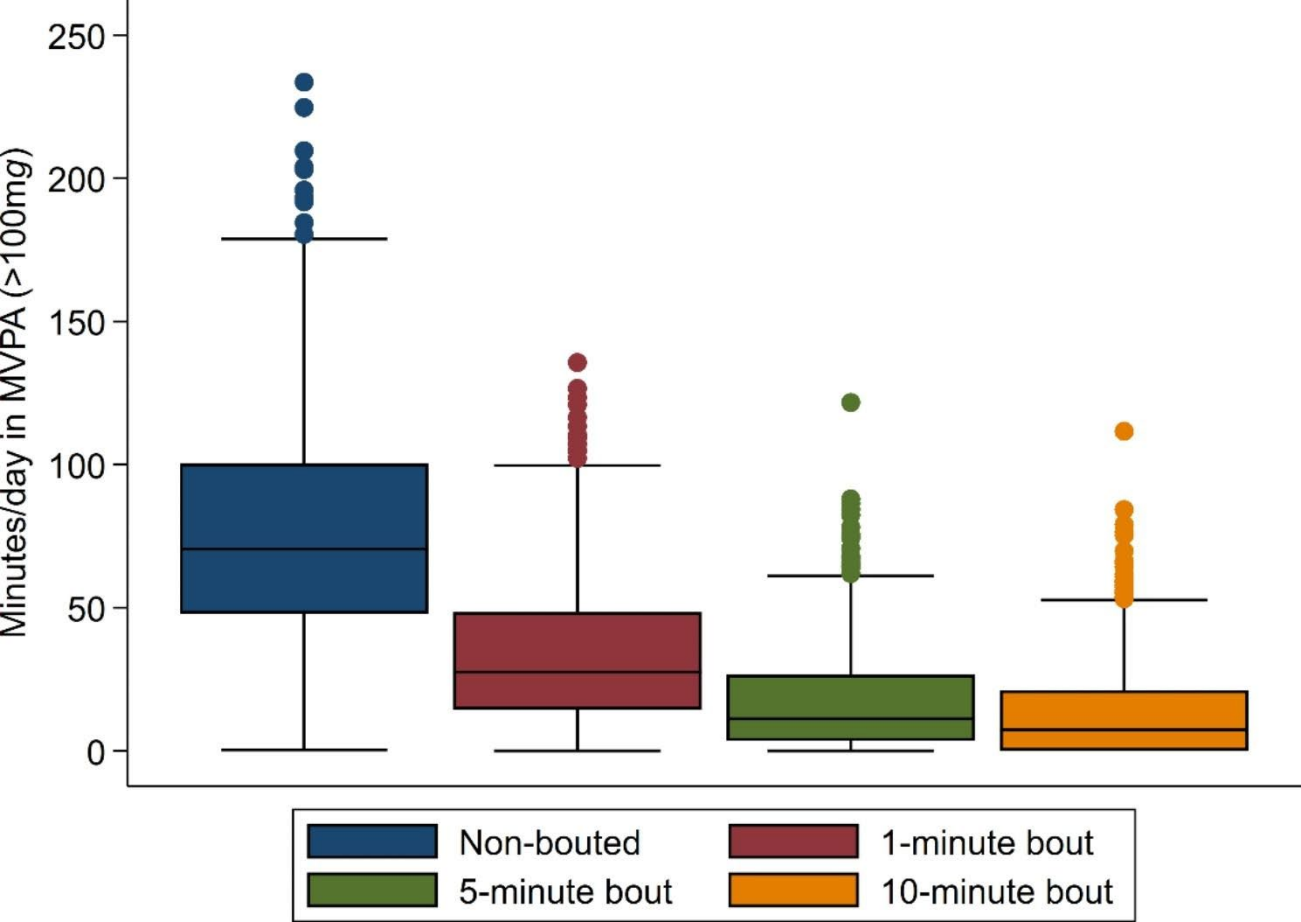
Box-and-whisker plot of average minutes per day in moderate-vigorous physical activity according to bout duration. Brisbane 2016 (N = 700)

The distribution of daily physical activity by levels of acceleration is presented in Fig. 1. Most time was spent in activities with an average acceleration between 50 and 99 m*g* (light intensity). Daily median duration of light intensity was 141 min. Of the total time spent in activities with acceleration ≥ 100 m*g* (68 min), two thirds were in activities with average acceleration between 100 and 149 m*g*. As shown in Fig. 2, median time spent in MVPA was 68 (25th -75th : 45–99) minutes/day when no bout criterion was used. This estimate decreased by approximately 60% for MVPA in bouts of 1-minute [Median: 26 (25th -75th : 12–46)]. When MVPA was estimated in bouts of 5-min and 10-min, the medians for MVPA were 10 (25th -75th : 3–24) and 8 (25th -75th : 0–19) minutes/day, respectively (Fig. 2).

Minutes in non-bouted MVPA and in bouts of 10-minutes, by sociodemographic variables, are presented in Table 2. Overall, the magnitude and direction of the associations between sociodemographic variables and MVPA varied by the bout criterion used. For example, total non-bouted MVPA did not differ by gender, but men accumulated more MVPA in bouts of 10 min than women. There were inverse associations between age and minutes in MVPA, and a positive association between income and MVPA, regardless of bout criteria. In the multivariate analyses, only age and occupation were associated with minutes in non-bouted MVPA and only gender and income were associated with MVPA in 10 min bouts. On average, labourers spent 25 min per day more in non-bouted MVPA than managers and professionals.

**Table 2:** Description of MVPA (minutes/day) non-bouted and in 10 min bouts, and associations with sociodemographic characteristics. Brisbane 2016 (N = 700)

	MVPA Non-bouted							MVPA 10 min-bouts					
**Variables**	**Mean (SD)**	**p** ^**a**^		**β** ^**b**^_**Crude**_ **(95%CI)**		**β** ^**b**^_**Adjusted**_ **(95%CI)** ^**c**^		**Median (IQR)**	**p** ^**a**^		**β** ^**b**^_**Crude**_ **(95%CI)**		**β** ^**b**^_**Adjusted**_ **(95%CI)** ^**c**^
Gender		0.153							0.001				
Men	79.9 (43.6)			Ref		Ref		10.5 (2.0; 25.2)			Ref		Ref
Women	75.4 (38.9)			-4.5 (-10.7; 1.7)		-0.4 (-7.0; 6.2)		5.7 (0.0; 17.7)			-4.8 (-7.9; -2.0)		-4.5 (-7.7; -1.3)
Age		< 0.001							0.004				
45–54	93.1 (43.1)			Ref		Ref		9.0 (2.3; 22.1)			Ref		Ref
55–64	78.9 (40.9)			-14.2 (-21.5; -7.0)		-14.2 (-21.9; -6.4)		8.5 (0.0; 23.1)			-0.4 (-3.9; 3.0)		-1.0 (-4.7; 2.7)
65+	62.4 (33.4)			-30.7 (-38.4; -23.0)		-25.4 (-35.0; -15.7)		4.7 (0.0; 17.7)			-4.3 (-7.9; -0.7)		-0.5 (-5.1; 4.2)
Education		0.927							0.025				
Year 12 or less	77.1 (42.3)			Ref		Ref		5.6 (0.0; 19.8)			Ref		Ref
Certificated/diploma	78.1 (42.0)			1.0 (-6.9; 8.9)		-2.3 (-10.3; 5.7)		8.4 (1.0; 20.7)			2.8 (-0.8; 6.4)		-0.2 (-4.1; 3.6)
Bachelor degree or higher	76.6 (39.1)			-0.5 (-7.8; 6.9)		0.0 (-8.4; 8.4)		8.4 (0.0; 22.1)			2.8 (-0.5; 6.1)		0.3 (-3.7; 4.4)
Income (per year)		0.003							< 0.001				
< $ 41,599	65.4 (39.1)			Ref		Ref		3.3 (0.0; 14.6)			Ref		Ref
$41,600 - $93,599	79.8 (44.9)			14.3 (6.0; 22.7)		4.1 (-4.6; 12.8)		6.2 (0.0; 21.9)			2.9 (-0.4; 6.2)		1.6 (-2.5; 5.8)
$93,600 +	81.1 (36.5)			15.7 (7.6; 23.8)		1.8 (-7.9; 11.5)		10.5 (2.2; 23.8)			7.3 (4.0; 10.5)		5.3 (0.6; 9.9)
Occupation ^d^		< 0.001							0.017				
Managers/ professionals	80.9 (37.2)			Ref		Ref		10.6 (2.0; 23.6)			Ref		Ref
Clerical/ administrative	76.1 (34.8)			-4.8 (-15.1; 5.5)		-5.1 (-16.6; 6.5)		6.6 (0.0; 19.8)			-4.1 (-9.1; 1.0)		-0.4 (-6.0; 5.1)
Community/ personal/ sales	91.3 (42.0)			10.4 (0.0; 20.9)		9.5 (-2.2; 21.3)		6.9 (1.8; 22.0)			-3.4 (-8.5; 1.7)		-0.4 (-6.1; 5.2)
Labourer/technician	101.1 (53.5)			20.3 (9.7; 30.8)		25.1 (12.9; 37.2)		3.3 (1.4; 28.4)			-3.9 (-9.1; 1.3)		-1.1 (-7.0; 4.7)
Retired	60.5 (33.3)			-20.4 (-28.1; -12.6)		-7.6 (-17.5; 2.4)		6.8 (0.0; 14.6)			-7.3 (-9.9; -3.5)		-2.2 (-7.0; 2.6)
Other/non paid work	72.5 (44.7)			-8.4 (-20.4; 3.5)		-5.9 (-19.3; 7.4)		8.0 (0.0; 21.3)			-3.9 (-9.8; 2.0)		-2.2 (-8.6; 4.2)

^a^ p-values for the comparison of average daily physical activity between groups

^b^ beta values represent the mean difference in average daily physical activity between groups (compared with the reference group)

^c^ mutually adjusted for other variables in the model

^d^ Labourer/technician: Technicians, trade, machinery operators, drivers and labourers

Proportions of individuals who did an average of at least one minute per day of vigorous activities (‘any VPA’) according to sociodemographic variables are presented in Table 3. Around one third of both men and women did at least one minute per day in vigorous intensity activities. This proportion was lowest among individuals 65 + years old, those with less than year 12 education, with annual income lower than $41,599, and among participants who were retired. In the adjusted analyses, only age was inversely associated with vigorous physical activity. Participants 65 + years were 57% less likely to do any vigorous intensity physical activity than those who were 45-54years.

**Table 3:** Proportion **any VPA (1-min bout)** by sociodemographic characteristics. Brisbane 2016 (n = 700)

Variables	%	p value ^a^		Crude		Adjusted ^b^
				**PR (95%CI)**		**PR (95%CI)**
Gender		0.535				
Men	33.3			1.00		1.00
Women	31.1			0.94 (0.75; 1.16)		1.02 (0.81; 1.28)
Age		< 0.001				
45–54	52.5			1.00		1.00
55–64	30.5			0.58 (0.47; 0.73)		0.65 (0.52; 0.83)
65+	17.6			0.34 (0.25; 0.46)		0.48 (0.33; 0.70)
Education		0.060				
Year 12 or less	26.3			1.00		1.00
Certificated/diploma	31.4			1.19 (0.88; 1.62)		1.05 (0.77; 1.43)
Bachelor degree or higher	36.3			1.38 (1.05; 1.81)		1.04 (0.76; 1.42)
Income (per year)		< 0.001				
< $ 41,599	24.8			1.00		1.00
$41,600 - $93,599	26.7			1.08 (0.76; 1.53)		0.85 (0.59; 1.24)
$93,600 +	43.1			1.74 (1.27; 2.37)		1.04 (0.77; 1.49)
Occupation ^c^		< 0.001				
Managers and professionals	43.9			1.00		1.00
Clerical and administrative	31.0			0.71 (0.49; 1.03)		0.75 (0.49; 1.13)
Community, personal and sales	33.8			0.77 (0.54; 1.11)		0.86 (0.57; 1.30)
Labourer/technicians	28.8			0.66 (0.44; 0.98)		0.83 (0.53; 1.29)
Retired	16.5			0.38 (0.26; 0.55)		0.60 (0.38; 0.96)
Other/non paid work	32.7			0.74 (0.49; 1.14)		0.80 (0.51; 1.26)

^a^ comparison of proportion between groups

^b^ mutually adjusted for other variables in the model

^c^ Labourer/technician: Technicians, trade, machinery operators, drivers and labourers

PR: prevalence ratio

## Discussion

This is the first study to describe physical activity estimated based on raw data from accelerometers in a cohort of Australian adults. In this unique large population-based cohort, our observations confirm that total physical activity varies by some sociodemographic characteristics, but with some unexpected findings which contrast previous research using self-report measures. In our study, the duration of total physical activity, as well as the magnitude and direction of the associations between sociodemographic characteristics and physical activity, depended on the bout criterion used in the analyses. Our analyses suggest that the commonly reported gender and socioeconomic differences in physical activity [26] emerge or are more pronounced when more prolonged physical activities are measured. Our findings also show that approximately one third of both men and women did at least one minute per day of vigorous intensity activity, however this was lower among those who were older and those who were retired.

The comparability of our findings with previous studies is limited by the scarcity of population-based studies with device-measures of physical activity during this life stage that used similar protocols. In our study, average acceleration per day was 23.8 mg. Previous studies that used similar protocols found similar estimates. In a sample of older adults (60 + years) Ramires and colleagues reported average daily acceleration of 23.4 m*g* in men and 23.1 m*g* in women [24]. Data from the UK Biobank show higher average acceleration among adults in the age range similar to that in our study [7].

Our estimates of time spent in activities of different intensity, as well as the direction and magnitude of differences in physical activity according to sociodemographic variables, varied according to bout duration. As has been demonstrated in previous studies [24], the estimates of average time in MVPA decrease with more restrictive bout criteria. These differences in physical activity estimates highlight important measurement issues, especially in relation to compliance with current physical activity guidelines which were developed based on evidence from self-report measures [3]. For example, in our study the proportion of respondents who technically met the physical activity recommendation of at least 150 minutes per week in MVPA was 95.1%, 59.3%, 32.2% and 24.2% when different bout criteria were used. This reinforces that caution is needed when using accelerometers to assess ‘prevalence’ of meeting physical activity guidelines. As current physical activity guidelines are based on self-reported data, it is erroneous to base estimates of compliance with guidelines using data from accelerometers. Our results also highlight the importance of detailing the methods used to manage accelerometer data, as different criteria for this “objective” method can produce different results.

Global estimates of self-reported physical activity in 142 countries show that women are less active than men in most countries. In Australia, the prevalence of physical inactivity assessed using self-report measures is approximately 30–40% higher in women than in men [26]. Our study, however, did not show consistent gender differences. Overall, there were no gender differences in average acceleration and non-bouted MVPA per day. These findings are similar to those from population-based studies in Norway, Sweden and the US, which have shown that mid-age women and men did not differ when overall physical activity levels were measured with accelerometers [27, 28]. Moreover, previous studies with self-report measures of physical activity have suggested that the gender gap in physical activity might not occur in mid-age and older adults [29]. However, in our study, women spent slightly less time than men in 10 min bouted MVPA. It may be that women accumulate more of their physical activity in brief bouts of incidental activities, whereas men may engage in more continuous physical activities. This highlights the advantage of this type of assessment, which is not well-captured in self-reported measures.

Our data confirm the well documented inverse association between physical activity and age. However, the magnitude of associations between age and MVPA were slightly attenuated in the adjusted models. Our findings could suggest that the association between retirement and physical activity is confounded by age, and that retirement does not necessarily explain the age differences in physical activity.

Socioeconomic position is often demonstrated as an important correlate of physical activity levels. Our findings showed that income was positively associated with physical activity, but education was not. Other studies with self-reported measures of physical activity have shown similar results [29, 30]. This may be partly explained by the extent to which different indicators of socioeconomic position may enable or constrain physical activity. Positive associations between income and physical activity and might represent access to resources such as health and sporting equipment and/or clubs and supervised exercise training. High income may also reflect more control over working conditions to enable discretionary time for physical activity. In contrast, education, which reflects knowledge attainment, can have a variable association with income and working conditions.

Adjusted analyses indicated that only age was inversely associated with vigorous physical activity: participants aged 65 + years were 57% less likely to do any vigorous intensity physical activity than those who were 45-54years. However, approximately one third of both men and women did at least one minute per day in vigorous intensity activities. This is important given previous research showing small amounts of high-intensity habitual physical activity, such as one minute per day, can be beneficial for health [10, 11]. This type of physical activity participation may provide more viable opportunities for people who are reluctant or disinterested in vigorous activity.

This study has several strengths. Physical activity was measured using accelerometry which is less susceptible to the biases associated with recall and social desirability intrinsic to self-reported measures [4]. The use of device measured MVPA accumulated in different bout lengths provides the opportunity to understand the potential importance of less structured activities (i.e. activities that were not sustained for at least 10 min), accumulated throughout the day, for health outcomes. The use of raw data is a strength because it allows comparability between studies, regardless of decisions about data processing [14]. The participants were part of a larger randomly selected population-based sample, which enabled us to examine a range of sociodemographic variables and contributes to the generalisability of results.

Some limitations of our study should be considered. This study was based on data from a subsample of the original cohort. Although the analytical sample is likely representative of Brisbane residents, there is slight over representation of individuals with high socioeconomic position, who tend to more active (based on self-report data) than more disadvantaged participants [17]. Hence physical activity levels in our study may be overestimated. By estimating physical activity using accelerometers, we were unable to provide the context or domains (e.g. leisure, transportation, work-based) of physical activity. Future studies could integrate objective measures of physical activity and self-report data for more description. Studies could also use GPS data to identify the location and type of physical activity. This study was conducted in one major metropolitan city in Australia, which may not generalise to other areas, in particular rural and remote locations. As the study participants were drawn from those who had a history of responding to the larger mail study, it may be that the participants were healthier and more interested in the study focus.

## Conclusion

Findings of this study have shown that accumulation of physical activity among mid-age older adults occurs mostly through activities of light to moderate intensity, in short bouts. These findings suggest substantial low levels of physical activity in older people and those with low income. These findings are important as they identify specific groups which should be targeted by public health interventions for increasing population levels of physical activity.

## Data Availability

The datasets generated and/or analysed during the current study are not publicly, but de-identified data are available from the corresponding author on reasonable request.

## References

[1] Sallis JF, Bull F, Guthold R, Heath GW, Inoue S, Kelly P (2016). Progress in physical activity over the Olympic quadrennium. Lancet.

[2] Varela AR, Pratt M, Powell K, Lee IM, Bauman A, Heath G (2017). Worldwide Surveillance, Policy, and Research on Physical Activity and Health: The Global Observatory for Physical Activity. J Phys Act Health.

[3] Bull FC, Al-Ansari SS, Biddle S, Borodulin K, Buman MP, Cardon G (2020). World Health Organization 2020 guidelines on physical activity and sedentary behaviour. Br J Sports Med.

[4] Warren JM, Ekelund U, Besson H, Mezzani A, Geladas N, Vanhees L (2010). Assessment of physical activity - a review of methodologies with reference to epidemiological research: a report of the exercise physiology section of the European Association of Cardiovascular Prevention and Rehabilitation. Eur J Cardiovasc Prev Rehabil.

[5] Brady R, Brown WJ, Hillsdon M, Mielke GI. Patterns of Accelerometer-measured Physical Activity and Health Outcomes in Adults: A Systematic Review. Med Sci Sports Exerc. 2022. Volume 54 - Issue 7 - p 1155-1166 doi: 10.1249/MSS.0000000000002900 10.1249/MSS.0000000000002900 35220369

[6] Troiano RP, McClain JJ, Brychta RJ, Chen KY (2014). Evolution of accelerometer methods for physical activity research. Br J Sports Med.

[7] Doherty A, Jackson D, Hammerla N, Plotz T, Olivier P, Granat MH (2017). Large Scale Population Assessment of Physical Activity Using Wrist Worn Accelerometers: The UK Biobank Study. PLoS ONE.

[8] Ekelund U, Dalene KE, Tarp J, Lee IM (2020). Physical activity and mortality: what is the dose response and how big is the effect?. Br J Sports Med.

[9] Menai M, van Hees VT, Elbaz A, Kivimaki M, Singh-Manoux A, Sabia S (2017). Accelerometer assessed moderate-to-vigorous physical activity and successful ageing: results from the Whitehall II study. Sci Rep.

[10] Ku PW, Hamer M, Liao Y, Hsueh MC, Chen LJ (2020). Device-measured light-intensity physical activity and mortality: A meta-analysis. Scand J Med Sci Sports.

[11] Stiles VH, Metcalf BS, Knapp KM, Rowlands AV (2017). A small amount of precisely measured high-intensity habitual physical activity predicts bone health in pre- and post-menopausal women in UK Biobank. Int J Epidemiol.

[12] Stamatakis E, Johnson NA, Powell L, Hamer M, Rangul V, Holtermann A. Short and sporadic bouts in the 2018 US physical activity guidelines: is high-intensity incidental physical activity the new HIIT? Br J Sports Med. 2019. 10.1136/bjsports-2018-100397 PMC6818666 30786998

[13] Migueles JH, Cadenas-Sanchez C, Ekelund U, Delisle Nystrom C, Mora-Gonzalez J, Lof M (2017). Accelerometer Data Collection and Processing Criteria to Assess Physical Activity and Other Outcomes: A Systematic Review and Practical Considerations. Sports Med.

[14] Migueles JH, Rowlands AV, Huber F, Sabia S, van Hees VT (2019). GGIR: A Research Community–Driven Open Source R Package for Generating Physical Activity and Sleep Outcomes From Multi-Day Raw Accelerometer Data. J Meas Phys Behav.

[15] da Silva IC, van Hees VT, Ramires VV, Knuth AG, Bielemann RM, Ekelund U (2014). Physical activity levels in three Brazilian birth cohorts as assessed with raw triaxial wrist accelerometry. Int J Epidemiol.

[16] Rowlands AV, Sherar LB, Fairclough SJ, Yates T, Edwardson CL, Harrington DM (2019). A data-driven, meaningful, easy to interpret, standardised accelerometer outcome variable for global surveillance. J Sci Med Sport.

[17] Burton NW, Haynes M, Wilson LA, Giles-Corti B, Oldenburg BF, Brown WJ (2009). HABITAT: A longitudinal multilevel study of physical activity change in mid-aged adults. BMC Public Health.

[18] Mielke GI, Burton NW, Turrell G, Brown WJ (2018). Temporal trends in sitting time by domain in a cohort of mid-age Australian men and women. Maturitas.

[19] Mielke GI, Bailey TG, Burton NW, Brown WJ (2020). Participation in sports/recreational activities and incidence of hypertension, diabetes, and obesity in adults. Scand J Med Sci Sports.

[20] Rosenberger ME, Haskell WL, Albinali F, Mota S, Nawyn J, Intille S (2013). Estimating activity and sedentary behavior from an accelerometer on the hip or wrist. Med Sci Sports Exerc.

[21] van Hees VT, Gorzelniak L, Dean Leon EC, Eder M, Pias M, Taherian S (2013). Separating movement and gravity components in an acceleration signal and implications for the assessment of human daily physical activity. PLoS ONE.

[22] van Hees VT, Fang Z, Langford J, Assah F, Mohammad A, da Silva IC (2014). Autocalibration of accelerometer data for free-living physical activity assessment using local gravity and temperature: an evaluation on four continents. J Appl Physiol (1985).

[23] Hildebrand M, VT VANH, Hansen BH, Ekelund U (2014). Age group comparability of raw accelerometer output from wrist- and hip-worn monitors. Med Sci Sports Exerc.

[24] Ramires VV, Wehrmeister FC, Bohm AW, Galliano L, Ekelund U, Brage S (2017). Physical activity levels objectively measured among older adults: a population-based study in a Southern city of Brazil. Int J Behav Nutr Phys Act.

[25] Mielke GI, Menezes AMB, da Silva BGC, Ekelund U, Crochemore-Silva I, Wehrmeister FC, et al. Associations between Device-measured Physical Activity and Cardiometabolic Health in the Transition to Early Adulthood. Med Sci Sports Exerc. 2021. 10.1249/MSS.0000000000002696 33966000

[26] Mielke GI, da Silva ICM, Kolbe-Alexander TL, Brown WJ (2018). Shifting the Physical Inactivity Curve Worldwide by Closing the Gender Gap. Sports Med.

[27] Hagstromer M, Troiano RP, Sjostrom M, Berrigan D (2010). Levels and patterns of objectively assessed physical activity–a comparison between Sweden and the United States. Am J Epidemiol.

[28] Hansen BH, Kolle E, Dyrstad SM, Holme I, Anderssen SA (2012). Accelerometer-determined physical activity in adults and older people. Med Sci Sports Exerc.

[29] da Silva ICM, Mielke GI, Bertoldi AD, Arrais PSD, Luiza VL, Mengue SS (2018). Overall and Leisure-Time Physical Activity Among Brazilian Adults: National Survey Based on the Global Physical Activity Questionnaire. J Phys Act Health.

[30] Turrell G, Haynes M, Burton NW, Giles-Corti B, Oldenburg B, Wilson LA (2010). Neighborhood disadvantage and physical activity: baseline results from the HABITAT multilevel longitudinal study. Ann Epidemiol.

